# Autonomous Robotic Surgery: Has the Future Arrived?

**DOI:** 10.7759/cureus.52243

**Published:** 2024-01-14

**Authors:** Yeisson Rivero-Moreno, Miguel Rodriguez, Paola Losada-Muñoz, Samantha Redden, Saiddys Lopez-Lezama, Andrea Vidal-Gallardo, Debbye Machado-Paled, Jesus Cordova Guilarte, Sheyla Teran-Quintero

**Affiliations:** 1 Department of Surgery, Universidad de Oriente, Barcelona, VEN; 2 Department of Surgery, Mercury Clinical Research, Houston, USA; 3 Department of Otolaryngology - Head and Neck Surgery, Baylor College of Medicine, Houston, USA; 4 Department of Surgery, Universidad de los Andes, Merida, VEN; 5 Department of Surgery, Universidad Católica de Honduras, Tegucigalpa, HND; 6 Department of Surgery, Universidad de Carabobo, Valencia, VEN

**Keywords:** clinical applications, ethical concerns, legal regulations, artificial intelligence, robotic surgery milestones, degrees of autonomy, autonomous robotic surgery

## Abstract

Autonomous robotic surgery represents a pioneering field dedicated to the integration of robotic systems with varying degrees of autonomy for the execution of surgical procedures. This paradigm shift is made possible by the progressive integration of artificial intelligence (AI) and machine learning (ML) into the realm of surgical interventions. While the majority of autonomous robotic systems remain in the experimental phase, a notable subset has successfully transitioned into clinical applications. Noteworthy procedures, such as venipuncture, hair implantations, intestinal anastomosis, total knee replacement, cochlear implant, radiosurgery, and knot tying, among others, exemplify the current capabilities of autonomous surgical systems. This review endeavors to comprehensively address facets of autonomous robotic surgery, commencing with a concise elucidation of fundamental concepts and traversing the pivotal milestones in the historical evolution of robotic surgery. This historical trajectory underscores the incremental assimilation of autonomous systems into surgical practices. This review aims to address topics related to autonomous robotic surgery, starting with a description of fundamental concepts and going through the milestones in robotic surgery history that also show the gradual incorporations of autonomous systems. It also includes a discussion of the key benefits and risks of this technology, the degrees of autonomy in surgical robots, their limitations, the current legal regulations governing their usage, and the main ethical concerns inherent to their nature.

## Introduction and background

Autonomous robotic surgery, removing the surgeon's hands, is a groundbreaking field that aims to introduce robotic systems capable of performing surgical procedures with a high degree of autonomy [1].

Autonomous control of surgical robotic platforms promises higher precision, intelligent maneuvers, and tissue damage avoidance. While most autonomous robotic systems are still experimental, some have reached clinical application. Research is ongoing to develop fully autonomous surgical systems that can perform complex tasks on deformable soft tissues, such as suturing and intestinal anastomosis, in an open surgical setting. Preliminary results have demonstrated that supervised autonomous procedures can outperform surgery performed by expert surgeons and robot-assisted approaches in terms of efficacy and consistency. These advancements in autonomous robotic surgery show the potential to improve surgical outcomes and accessibility to optimized techniques [2].

The development of autonomous actions in surgery has been met with skepticism from some surgeons. However, advances in artificial intelligence (AI) and robotics have opened possibilities for more autonomous actions in surgical procedures. The lack of haptics, or the sense of touch, has been a barrier to the broader adoption of robotic surgery, the true potential of robotics is being recognized, and the integration of AI by the surgical community is becoming more critical than ever [1].

Surgeons are encouraged to interpret and steer these technologies toward optimal patient care and net social benefit, leveraging distinctively human qualities such as creativity, altruism, and moral deliberation. By embracing these technologies, surgeons can free up time to focus on critical aspects of patient care and patient interaction [3].

## Review

Fundamental concepts: artificial intelligence (AI), machine learning (ML), and deep learning (DL) and its application

The two most crucial components of effective patient care are expertise and knowledge. The more patients we treat and the more information we have, the better patient care we can offer. Typically, this happens over time, so doctors gain experience and knowledge while treating patients and through ongoing education. We are better equipped to make knowledge-based decisions the more experience and data (information analysis) we possess. However, time limits are the primary reason why the human mind is limited in its ability to process vast volumes of material. Nonetheless, an enormous quantity of patient data may be accessible, collected, and saved for processing in the age of silicon chips. The foundation ofartificial Intelligence (AI) is using these massive data banks and changing them to gain experience. By using algorithms, computer software may therefore learn substantially more expertise in a far shorter length of time than human subjects can in their lifetime. Consequently, AI refers to the capability of a machine or a program to mimic human intelligence and perform tasks such as reasoning, problem-solving, and learning based on the data provided [4].

Nowadays, AI permeates many aspects of our daily lives, including computer games, automated public transit, personal assistants (such as Siri, Alexa, and Google Assistant), and flying. AI has more recently started to be used in medicine to enhance patient care by accelerating procedures and obtaining higher accuracy, paving the way for the provision of improved healthcare as a whole [4].

ChatGPT is one of the AI tools that has recently attracted interest in the medical industry. OpenAI's ChatGPT is a sophisticated language model that uses deep learning (DL) methods to generate responses to natural language inputs that resemble those of a human. Medical practitioners may find ChatGPT useful in disease diagnosis, therapy recommendation, and outcome prediction. By providing students with pertinent and educational answers to their questions, it might also help with medical education. Because the model can produce replies that are similar to those of a human in response to natural language inputs, ChatGPT has demonstrated encouraging outcomes in a number of medical domains. This can help medical professionals make decisions. However, using ChatGPT in medical settings also brings up moral questions about openness and responsibility [5].

Machine learning (ML) is a subset of AI that uses the data and history of information provided to the program repeatedly and recognizes patterns to improve the performance of tasks according to that data. It is also seen as a computer's capacity to learn from experience, that is, to adjust how it processes data on the basis of newly acquired information. Considered as an application of AI, ML algorithms include supervised, unsupervised, and reinforcement learning. Today, ML is practically used in biometric attendance, face recognition in the healthcare sector, banks, retailers, travel, amazon, amazon Alexa, and voice helps in various applications of computers [6].

On the other hand,* *deep learning is a type of ML directly inspired by the architecture of neurons in the human brain. An artificial neural network is made up of multiple layers, through which data is processed. This is what allows the machine to "go deeper" in its learning, identifying connections and altering input data to achieve the best results [6].

In DL, various datasets are simultaneously considered, undergoing multiple evaluations and reprocessing for successive assessments. This iterative process continues through different layers until an ultimate output is attained. Each evaluation occurs within a distinct layer, building upon the output of the preceding layer. These computation layers are referred to as hidden layers due to the invisibility of their inputs and outputs. For instance, when analyzing a colonoscopy image to detect polyps, the image undergoes initial multiplication. Subsequently, the image is subjected to diverse filters, each producing a score that is then transmitted to subsequent layers of filters, such as color and edge detection filters. This layered workflow persists through multiple stages, hence the term "deep learning." Each filter generates an output score, serving as the input score for the subsequent layer, culminating in a final result, which could be a diagnostic outcome or the identification of a polyp in an image [4].

ML and DL represent the virtual branch that uses mathematical algorithms for promoting learning via experience as already discussed. Medical devices and other AI-operated physical objects are examples of physical AI. Robot companions have been introduced for the geriatric population experiencing cognitive decline and decreased mobility. AI is currently used to evaluate human performance in rehabilitation systems. Also, nanorobots have been made to regulate and monitor the drug delivery system in humans. In this way, AI and ML assist in evaluating and measuring treatment effectiveness [7].

The creation of a DL algorithm by Gulshan et al. to identify diabetic retinopathy from retinal fundus photos is a modern medical example of DL. Using 128,175 retinal photos that had previously been examined by ophthalmologists as their training set, they created a DL algorithm capable of analyzing fresh images and achieved a 97.5% sensitivity and 93.4% specificity in identifying diabetic retinopathy [8].

Such results have significant implications for screening and service delivery if they are sustained and validated repeatedly in real-world situations.

In certain clinical fields, the application of DL has occurred more quickly due to the conditions (digitization and high volume of data) that allow it, as occurs in radiology, radiotherapy, pathology, ophthalmology, dermatology, and image-guided surgery, while in others, progress has been paused due to limitations and it has not even started [9].

Artificial intelligence and machine learning in the surgical field

AI in surgery involves autonomous movements. Fortunately, more surgeons are becoming interested in technology and the possibility of autonomous actions in procedures such as general surgery, endoscopy, and interventional radiology as the field of robotics in surgery has advanced [1].

The two primary areas in which ML has been used most frequently in the surgical sector are robotics and decision assistance. Similar to medicine and diagnostics, ML decision support systems are becoming more and more prevalent for diagnosing conditions, predicting outcomes, and identifying surgical candidates as well as possible postoperative problems [10].

AI-operated machines assist in preoperative planning and visualization of patient anatomy, thereby improving surgical precision, safety, and training for the benefit of patients and the healthcare community [11].

All of these factors and innovations in minimally invasive surgery fit into traditional S-shaped curves that have three phases: (1) new technology introduction, (2) achieving a performance advantage over current standards, and (3) reaching a performance plateau, after which an innovation with more machine autonomy and less human influence is added or replaced [3].

Surgeons may suffer from exhaustion, imprecision, and fluctuations in technical proficiency, which could affect their patients. Technological developments in minimally invasive surgery enhance the manual dexterity skills of surgeons and hold promise for autonomous robotic surgery [3].

Several studies have already demonstrated the superiority in accuracy, sensitivity, and specificity of AI and ML applications in the surgical field.

A systematic review that included any clinical studies that present the diagnostic and prognostic accuracies of machine learning models in the clinical setting of plastic surgery demonstrated that the clinical utility of these algorithms was to assist clinicians in diagnosis prediction, outcome prediction, and preoperative planning with a mean accuracy of 88.80%, 86.11%, and 80.28%, respectively [12].

A prognostic study that was conducted among 1,477,561 patients undergoing surgery at 20 community and tertiary care hospitals in the University of Pittsburgh Medical Center (UPMC) health network aimed to evaluate the accuracy of an automated machine learning model in the identification of patients at high risk of adverse outcomes (postoperative mortality and major adverse cardiac and cerebrovascular events at 30 days) from surgery using only data in the electronic health record. Accuracy was compared between the UPMC model and the National Surgical Quality Improvement Program (NSQIP) surgical risk calculator for predicting mortality. The model outperformed the NSQIP tool with a specificity of 87% and an accuracy of 85% [13].

Ensemble ML using random forests, neural networks, and lasso regression was able to predict patient lung cancer staging using only International Classification of Diseases (ICD)-9 claims data with 93% sensitivity, 92% specificity, and 93% accuracy, outperforming a decision tree approach based solely on clinical guidelines (53% sensitivity, 89% specificity, and 72% accuracy) by analyzing patterns of diagnostic and therapeutic data (including surgical resection) in the Surveillance, Epidemiology, and End Results cancer registry and comparing data to Medicare claims [14].

The calculation capacity of machines is much faster and more precise than that of humans, but there are non-numerical variables related to non-tangible aspects that machines are not yet able to include in their decision processes [15].

History and development of autonomous robotic surgery

Autonomous robotic surgery has been developing progressively, incorporating functions into the existing robotic systems that increased their degree of autonomy. Therefore, in some cases, it is difficult to give an exact date for the first autonomous procedure in a specific field. In Table 1, we summarize the principal milestones in robotic surgery including the beginning of autonomous systems and pointing out the level of autonomy according to Yang et al. [16].

**Table 1:** Milestones in robotic surgery FDA: US Food and Drug Administration, AESOP: Automated Endoscopic System for Optimal Positioning, TORS: transoral robotic surgery, STAR: Smart Tissue Autonomous Robot Note: This list does not include the total milestones in robotic surgery history but gives an overview of the most important facts described in the current literature. Sources: [17, 18]

Year	Milestone	Level of autonomy
1985	The first surgical robot was used to perform brain biopsies by PUMA 200 (Westinghouse Electric, Pittsburgh, PA).	Level 0
1989	First urologic robot for transurethral resection of the prostate (PROBOT).	Level 1
1992	ROBODOC (Integrated Surgical Solutions, Inc. and IBM) is used to prepare a femur for hip replacement in human subjects.	Level 0
1994	First commercially available robot approved by the FDA (AESOP). It aims to manage laparoscopic procedures using voice control, allowing greater flexibility.	Level 0
1998	Zeus system (Computer Motion Inc.) approved by the FDA and made commercially available.	Level 0
2000	Da Vinci system approved by the FDA for general surgery (Intuitive Surgical Inc.).	Level 0
2001	The first robot-assisted radical prostatectomy is performed.	Level 1
2001	The CyberKnife radiosurgical robotic system (Accuracy, Madison, WI) received FDA approval for radiosurgery.	Level 2-3
2000	Total knee replacement arthropathy with a computer-assisted orthopedic planning system for ROBODOC.	Level 2
2005	Da Vinci® robot made the first TORS resections of the base of tongue neoplasms.	Level 2
2005	The ARTAS Robotic Hair Restoration System was developed.	Level 3
2006	Robotic surgery started to be used in gynecology.	Level 1
2009	Veebot, the first robot for autonomous blood sampling, is launched on the market.	Level 3
2012	The first total knee arthroplasty was performed by TSolution.	Level 3
2016	The STAR performed the first autonomous procedure: bowel anastomosis.	Level 3
2016	An ETH Zurich team described a hydrogel microrobot that propels itself through viscous solutions.	Level 3

Levels of autonomy in robotic surgery

The first attempt to organize research on autonomous robotic surgery was undertaken in 2017 by Yang et al., who divided the autonomy that a surgical robot may achieve into six stages [16,19].

Level 0

The da Vinci system from Intuitive Surgical established the paradigm of transparent teleoperation in 2000. In this system, the patient's surgical tools precisely mimic the movements of the surgeon on the control interface. The surgeon alone is in charge of controlling the robot's motion; no supports or limitations are given [19].

Level 1

In order to assist or direct the surgeon in carrying out a certain task, robots can communicate with them. Either virtual fixtures to improve the view of the surgical site or active limitations to direct the surgeon's motion are the forms of help offered. The majority of level 1 research platforms gather a small amount of data that is generally low-complexity and pertains to the robot, the surgeon, or the target tissues. The three primary enabling technologies for level 1 autonomy are tissue interface sensing, tool tracking, and eye tracking [19].

Level 2

Robots are competent to complete particular surgical activities according to the guidelines given by the physician. For the length of the task, the robot's control transitions from the human operator to the machine [19].

An illustration of this is tip retroflection in magnetic colonoscopy, which enables the operator to examine a greater area of the colon during a colonoscopy due to retrograde vision. However, predicting how to alter the controlling magnetic field and field gradient to achieve the desired motion at the endoscope tip is very difficult for a human operator to perform. Therefore, Slawinski et al. proposed an autonomous algorithm that tracks the endoscope tip's pose in real time and modifies the external driving magnet's pose accordingly to achieve retroflection. The robotic colonoscopy platform typically operates in transparent teleoperation with active constraints (level 1), and the algorithm activates when the operator needs retroflection [20].

Using visual markers to determine the flap-grasping location and fuzzy logic to carry out the motion, Nagy et al.'s investigation using a da Vinci Research Kit proposed a tissue retraction system. Automated procedures in robotic surgery include tissue retraction, ablation, and suturing (from needle insertion to knot tying) [21].

Kang and Wen were the first to look at robotic knot tying in minimally invasive surgery. They have created EndoBot, a specialized robotic system. The robot carries out tasks autonomously and in a supervised manner. Despite the fact that the studies are encouraging, it appears that the robot follows a hard-wired policy, which means that it repeats the same motion without considering the potential of carrying out the same task with different instrument placements [22].

An autonomous micro-drilling robot has been built by Taylor et al. to perform cochleostomy, a fundamental step in cochlear implantation that involves drilling a hole in the cochlea's outer wall through which the electrode implant is put. With the drill pointing in the direction of the intended trajectory, the surgeon moves the arm into the proper position. The drill then automatically makes the hole while keeping the endosteal membrane intact. The arm is then locked, and a knife is used to open the hole [23].

Level 3

Perceptual abilities are given to robots so they can plan and carry out certain tasks, comprehend the surgical setting, and alter the plan as they go. Comparable to level 2, robot control shifts from the surgeon to the device while the work is being completed [19].

Examples of this technology include autonomous navigation of flexible endoscopic robots in unstructured environments as the one proposed by Martin et al. that uses magnetic field sensing, robotic control, and real-time image processing to enable autonomous maneuvering during colonoscopy [24]. More examples are given in the next section.

Level 4

The robot is capable of interpreting preoperative and intraoperative data, creating an interventional plan consisting of a series of actions, carrying out this plan on its own, and making adjustments to the plan as needed. In the discrete control paradigm, the system is overseen by a surgeon. We can easily see how these systems would improve the way healthcare is delivered, even though specific examples are not yet available. For instance, they could be used for the intelligent removal of cancerous tissue, which would involve registering with preoperative imaging, adapting the plan based on real-time data, and ablation of cancer while sparing as much healthy tissue as possible [19].

Level 5

Robots are capable of performing surgery without assistance from a human. Since no systems have reached this level, it is not covered in this review [19].

**Figure 1:**
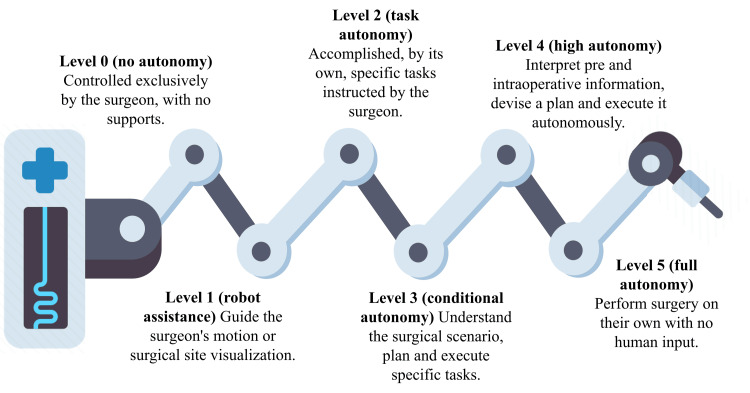
Levels of autonomy in robotic surgery according to Yang et al.'s classification Source of the image: authors Source: [16]

Figure 1 provides a basic explanation of this degree of autonomy.

Current autonomous robotic surgery interventions

The following examples describe other cases of autonomous robotic surgery in level 3.

The Smart Tissue Autonomous Robot (STAR) developed by Axel Kriger matched and even outperformed human surgeons in ex vivo or in vivo bowel anastomosis. It is an autonomous robot that needs only human approval of the plan but performs the procedure independently. The first surgery by STAR was performed on June 11, 2016. When used on the phantom bowels, STAR made fewer mistakes as compared to human surgeons. Also, the flow of the viscous fluid was more laminar and smoother through the bowel reconstructed by STAR, which shows efficacy. Based on the thickness and structure of the tissue, STAR creates a plan for suture insertion after the surgeon manually exposes the tissue edges. STAR autonomously sews the tissue together after the human operator gives the go-ahead. STAR asks the surgeon whether a new surgical plan is necessary if the tissue deforms or travels past a certain threshold. Until the robot completes the full procedure, this process is repeated [25].

While current results on advanced suturing are extremely encouraging, they are limited to anatomical phantoms or ex vivo tissue models. As the approach is translated to more realistic scenarios, the performance of the suturing robot may be heavily affected. From the small amount of literature available, it is clear that full autonomous suturing is still far from being commercially available [19].

Another autonomous robotic system that can carve bone on its own in accordance with a predetermined plan is called TSolution One. These days, it is employed for bone drilling, particularly in hip and knee replacement procedures. In August 2012, TSolution One carried out the first total knee arthroplasty (TKA) at Busan Centum Hospital in Korea. The existing technology cannot distinguish between different types of tissue; instead, it executes scheduled cuts inside the predefined 3D area. To avoid damaging soft tissues during the process, the surgeon must transfer those tissues out of the miller's path. Because long-term survival and outcome data are lacking, the cost-effectiveness of TSolution One® TKA has not been established [26].

Veebot is one of numerous automated blood sample systems. A patient places his or her arm through an archway over a padded table to operate the Veebot device. An inflatable cuff inside the archway tightens around the arm, keeping it in position and reducing blood flow to enhance the visibility of the veins. The inner elbow is illuminated for a camera by an infrared light; software compares the image from the camera with a model of vein anatomy and identifies a likely vein. Using ultrasonography, the vein is inspected to ensure that it is sufficiently large and has enough blood flowing through it. After that, the robot inserts the needle after aligning it. The technician just needs to attach the proper test tube or IV bag during the about one-minute procedure. As of right now, Veebot's machine can roughly identify the ideal vein to target 83% of the time, which is comparable to human performance [27].

The most recent advancement in hair restoration surgery is the robotic graft harvesting device known as the ARTAS system (Restoration Robotics Inc.). For the purpose of harvesting follicular units, this system employs the follicular unit extraction/follicular isolation approach, which is especially well adapted to the capabilities of robotic technology. The follicular units are harvested using a blunt dissecting punch and a dual-chamber needle after the donor area's pictures have been analyzed by the ARTAS system [28].

With its most sophisticated autonomous capabilities, the CyberKnife robot (Accuracy Inc.) performs radiosurgery for malignancies of the brain and spine while under human supervision. The CyberKnife system locates tumors using stereotactic concepts. Frequent acquisition of real-time orthogonal images of the patient during treatment allows the system to detect and automatically adjust for slight variations in the patient's posture [29].

Limitations in autonomous robotic surgery

Autonomous robotic surgery, despite its potential benefits, faces several limitations that need to be addressed. One of the main challenges is the complexity of handling highly intricate surgical procedures that demand adaptive decision-making in real time. While autonomous robots excel in preprogrammed and repetitive tasks, their autonomy is tested by the variability of anatomy and unexpected situations that may arise during surgery. Another limitation lies in their perception and feedback capabilities. Autonomous robots heavily rely on sensors and perception to interpret the surgical environment, but the quality of sensor data and limited tactile feedback can impede precise decision-making and real-time adaptation during surgical procedures. Moreover, autonomous surgical robots may struggle to adapt to unforeseen surgical scenarios or encounter complex situations not encountered during their training. The lack of experience in such new scenarios can impact their overall performance and efficacy [30].

The introduction of autonomous surgical robots also raises ethical and legal questions surrounding responsibility and accountability in the event of errors or malfunctions. Determining who is accountable in complex situations can be challenging and requires careful consideration [31]. Training and validating autonomous surgical robots is a demanding process that necessitates extensive data and rigorous testing to demonstrate their safety and accuracy. Insufficient data or validation can undermine their clinical reliability, making thorough assessment crucial before their widespread adoption. Additionally, the cost associated with developing, acquiring, and implementing autonomous surgical robots is often high, limiting their availability and accessibility, particularly in hospitals or regions with limited resources [19].

Addressing these limitations through ongoing research and technological advancements is vital to harness the full potential of autonomous robotic surgery in improving patient outcomes and advancing the field of surgery.

Legal and ethical considerations on autonomous robotic surgery

Regulations are fundamental to provide confidence, transparency, and safety in every procedure performed in healthcare. The literature on this subject is few and primarily theoretical because this is such a new and rapidly developing field of study. Three components of duty are highlighted in an intriguing ethical and legal viewpoint by O'Sullivan et al. [32].

Accountability

This refers to the capacity to explain choices, which declines with system complexity and may be remedied by combining recording black boxes with explainable artificial intelligence [32].

Liability

Even if the robot is autonomous, it may not be held accountable under current law for its deeds or inactions if they cause harm. Therefore, it cannot be mandated to pay the victim's compensation. In this instance, the harm a surgical robot causes to a patient is attributed to the manufacturer (in the event that the robot has a manufacturing defect), the operator (in the event that the robot's use is involved or a medical error has been made), or the person in charge of performing maintenance or making adjustments to the robot (in the event that the harm is the result of the robot's failure) [32].

Culpability

Culpability(the possibility of punishment) constitutes the most complex topic and could pose a significant legal and ethical barrier that influences the role of surgeons. A robot or artificial intelligence program cannot be held accountable for its actions in any way since it lacks conscience, free choice, or freedom in any other sense that is currently recognized by the legal system. It does not understand these ideas. Autonomous robots will be held accountable by those who produce, market, own, and use them, as well as by the laws and regulations that govern their use [32].

Current regulations of autonomous surgical robots

In the regulatory field, several important government agencies such as the British Medicines and Healthcare Products Regulatory Agency, the US FDA, and the German Federal Institute for Drugs and Medical Devices do not have specific legal frameworks for robots with autonomous actions [19].

Documents such as the EURON Roboethics Roadmap, which connects regulation, ethics, and standards, discussed the principles of robotics and suggests that autonomous robots should be human-monitored instead of fully independent, especially in medical diagnoses and surgical decisions that might be risky [33].

In the European Parliament resolution of 16 February 2017, with recommendations to the Commission on Civil Law Rules on Robotics, regarding medical robots, they stated that it is vital to respect the principle of the supervised autonomy of robots, whereby the initial planning of treatment and the final decision regarding its execution will always remain with a human surgeon [34].

Robotic systems with high degrees of autonomy are expected to make critical therapeutic choices in the future. This might create a new regulatory dilemma because medical organizations, not government agencies such as the FDA, are normally in charge of regulating medical practice due to legal limitations. These organizations, however, lack the technical know-how to assess these intricate and quickly developing technologies [19].

What if something goes wrong?

The surgeon retains total control because the machine does not make decisions. Due to this feature, Intuitive Surgical has been able to assert that, in the event of a technical malfunction, the surgeon bears full responsibility for the procedure. As a result, two of the more than 3,000 cases filed against the business up to 2016 have gone to trial. Despite the fact that Intuitive Surgical has been accused in multiple lawsuits of failing to provide medical staff members with the required training, all of those cases have been settled [19].

In a survey with more than 10,900 responses from all around the world, a dilemma was demonstrated among respondents on who to blame when harm is caused by a fully autonomous surgical robotic system. Importantly, it also showed that the surgeon is ascribed blame even when they have had no role in decision-making, which adds weight to concerns that human operators could act as "moral crumple zones" and bear the brunt of legal responsibility when a complex autonomous system causes harm [35].

Will the surgeons be replaced by robots?

Axel Krieger, the creator of the STAR system, believed that surgical robots were not intended to completely replace surgeons in the operating room. STAR and other autonomous robots are meant to work side by side with surgeons in the surgical workflow, helping them accomplish more accurate and repetitive tasks and ultimately increasing surgical consistency from patient to patient [36].

The American College of Surgeons published a report in June 2023 explaining how artificial intelligence is poised to "revolutionize" surgery. The majority of studies demonstrate that AI scan interpretation is more reliable and accurate than radiologists', frequently identifying tiny, seldom spots in the pictures. AI is meant to assist radiologists in finding a needle in a haystack, not to replace them. The same holds true for medical procedures. The majority of experts in robotic surgery and AI tend to concur that it is unlikely that human surgeons would ever be entirely replaced by an AI-controlled surgical robot. AI is meant to support, not replace, a surgeon's ability to make decisions and carry them out [37].

## Conclusions

In the near future, robotic technology will transform the surgical field. Robots now possess autonomous and semi-autonomous modes because of the development of new capabilities made possible by AI, machine learning, and deep learning. More research and development are being done on this autonomy in a variety of surgical procedures, from those that are now being utilized, such as cochlear implants, to experimental techniques such as fully autonomous intestinal anastomosis. High-level autonomous features are replacing the low-level automation of the first medical robots in terms of task complexity. With few official restrictions and very contentious ethical questions, the legal and ethical ramifications of autonomous activities by robots remain a topic of discussion.
